# The 2D : 4D Digit Ratio as a Biomarker for Autism Spectrum Disorder

**DOI:** 10.1155/2017/1048302

**Published:** 2017-07-19

**Authors:** M. Mackus, D. de Kruijff, L. S. Otten, A. D. Kraneveld, J. Garssen, J. C. Verster

**Affiliations:** ^1^Division of Pharmacology, Utrecht University, Utrecht, Netherlands; ^2^Institute for Risk Assessment Sciences (IRAS), Utrecht University, Utrecht, Netherlands; ^3^Nutricia Research, Utrecht, Netherlands; ^4^Centre for Human Psychopharmacology, Swinburne University, Melbourne, VIC, Australia

## Abstract

It has been suggested that the second (2D, index finger) to fourth (4D, ring finger) digit ratio, 2D : 4D, may be a biomarker for the risk of developing autism. The aim of the current study was to determine the usefulness of the 2D : 4D digit ratio as biomarker for autistic traits. *N* = 401 healthy young volunteers participated in the study. For both hands, digit lengths were measured using digital Vernier calipers. In addition to demographics, the Autism Spectrum Quotient (AQ) questionnaire was completed, comprised of five subscales, assessing “social insights and behavior,” “attention switching,” “communication,” “imagination,” and “attention to detail.” Overall, no significant correlations were observed between the AQ total score, its subscales, and the 2D : 4D digit ratio. For women, the left hand 2D : 4D digit ratio correlated significantly with the subscale score “communication” (*r* = −0.142; *p* = 0.036). For men, a significant positive correlation was found between the left 2D : 4D digit ratio and the total AQ score (*r* = 0.157; *p* = 0.042) and AQ subscale “attention switching” (*r* = 0.182; *p* = 0.017). In conclusion, gender specific associations between the 2D : 4D digit ratio and specific autism traits were observed, which were stronger in men than in women. Future studies should be conducted in patients that are formally diagnosed with autism.

## 1. Introduction

The second (2D, index finger) to fourth (4D, ring finger) digit ratio, 2D : 4D, has been suggested as a predictor of disease predisposition [1]. More specifically, the 2D : 4D digit ratio might be a useful predictor of fertility, differentiation pattern of the central nervous system (CNS), and the expression of particular adult-onset diseases, such as immune dysfunction, myocardial infarction, and breast cancer [1]. For example, low digit ratios are associated with increased fertility and vice versa.

Relative digit lengths are established in the 14th week of fetal development [2]. Low 2D : 4D digit ratios (2D < 4D) are associated with high levels of fetal testosterone, relative to fetal estrogen levels. In contrast, high values of digit ratios (2D > 4D) are associated with high fetal estrogen levels, relative to fetal testosterone levels [3]. This sexually dimorphic difference is most strongly expressed on the right side of the body [4]. Consequently, sexual traits can be most reliably correlated with the right digit ratio [3, 5]. This is distinguished between two ranges of digit ratios; individuals with digit ratios higher than 1.00 are defined as “dove” type, and individuals with digit ratios lower than 1.00 are defined as “hawk” type. As a consequence of varying concentrations of prenatal testosterone, digit ratios are said to be sexually dimorphic, meaning that this ratio is different in men and women. Males tend to have lower digit ratios than females [6, 7]. Digit ratios have already been suggested to correlate with various sexually dimorphic traits. The best-known association with the digit ratio is the masculinity of an individual, especially in men. Previous studies have found a direct relationship between male digit ratios and traits depending on pubertal and adult testosterone levels [8].

Interestingly, evidence is found for testosterone having a major impact on the differentiation of the CNS [9]. An important implication is the correlation of high prenatal levels of testosterone and the development of traits of autism spectrum disorder (ASD): since low digit ratios are associated with high levels of prenatal testosterone, these ratios are suggested to predict an elevated expression of autistic traits [10–12]. A meta-analysis of seven studies by Hönekopp compared the 2D : 4D digit ratio in both individuals diagnosed with ASD and unaffected controls [11]. This analysis showed that the digit ratio is on average lowered by 0.5–0.6 SD in individuals diagnosed with ASD. Measures of empathizing and systemizing did not show a relationship with the digit ratio neither in ASD-affected nor in healthy controls. This may be explained by the small fraction of individuals that received prenatal testosterone levels being considerably above the male average required to see such relevant effects in behavior. A meta-analysis by Teatero and Netley extends the results found by Hönekopp. The analysis of twenty-five studies found that ASD individuals tend to have their digit ratios lowered by 0.10–0.77 SDs, and systemizing and empathizing not to be correlated with these ratios [12].

Testosterone is suggested to inhibit the growth of certain areas of the left hemisphere and on the other hand to stimulate the growth of the same areas in the right hemisphere [9]. In addition, autism has been described as extreme manifestation of certain sexually dimorphic traits and is also defined as the consequence of an “extreme male brain” [13, 14]. This phenomenon is also called hypermasculinization and is found in autistic children. Hypermasculinization is a direct consequence of relative high testosterone levels in the fetal stage and is being linked to lower digit ratios [15]. Thus, the digit ratio could serve as a useful biomarker for the development of autistic traits.

It is important to further investigate whether the 2D : 4D digit ratio is useful as noninvasive biomarker for ASD. Diagnosis of ASD at a young age will enable early intervention, and better outcomes are proven to be a direct consequence of early diagnosis [16, 17]. Addressing the risk for developing disruptive symptoms such as communication problems at an early age may prevent or reduce their expression in later life [18]. Therefore, the aim of the present study was to examine the relationship between the presence and severity of autistic traits and the 2D : 4D digit ratio in young adults between 18 and 30 years old.

## 2. Methods

Students of Utrecht University, Netherlands, 18 to 30 years old, were recruited to participate in this study by completing a survey. Informed consent was obtained from all participants; no medical ethics approval was required to conduct this study [19].

In addition to demographic data, the 50-item adult Autism Spectrum Quotient (AQ) was completed as a quantitative measure of autistic traits [20]. Subjects had to rate to what extent they agree or disagree with statements on personal preferences and habits on a 4-point Likert scale (answering possibilities: “definitely agree,” “slightly agree,” “slightly disagree,” and “definitely disagree”). The AQ has 5 subscales, assessing the domains communication, social insights and behavior, imagination, attention to detail, and attention switching. A high score on a subscale implies an increased likelihood of possessing the corresponding autistic trait.

The length of the index (2D) and ring (4D) finger on both left and right hand were measured using a digital Vernier caliper. The total length of these digits was quantified from the middle of the basal crease to the tip of the finger and was determined in millimeters, with a recording accuracy of 0.01 mm. To optimize measuring precision, participants were asked to open their hands, laying them down on a flat surface, without overstretching them. All finger length measurements were conducted by the same researcher (MM), to control for between measurement errors.

IBM SPSS statistics version 24 was used for data analysis. 2D : 4D digit ratios were correlated with the total AQ score and its subscales. AQ scores were compared between those with a ratio greater than 1.00 (dove-type personality) and those with a digit ratio smaller than 1.00 (hawk-type personality). Analyses were conducted for the whole group and for men and women separately.

## 3. Results

*N* = 401 subjects participated in this study, with a mean (SD) age of 20.5 (2.3) years. The mean (SD) total AQ score was 102.2 (13.2). The mean left digit ratio (SD) was 0.988 (0.036), and the mean (SD) right digit ratio was 0.992 (0.034). The total AQ score was significantly higher in men compared to women (*p* = 0.000). When compared to women, men scored significantly higher on the subscales “social insights and behavior” (*p* = 0.010), “attention switching” (*p* = 0.018), “communication” (*p* = 0.000), and “imagination” (*p* = 0.000). Demographics for the group as a whole, and according to gender, are summarized in Table 1.

Overall (men and women combined), digit ratios of both hands did not significantly correlate with the total AQ score and the separate subscales. In both dove-type and hawk-type subjects, both the left hand and right hand 2D : 4D digit ratio did not significantly correlate with the total AQ score, nor with any of the AQ subscales.

### 3.1. Gender Differences

The right hand 2D : 4D digit ratio was significantly higher in men than in women (*p* = 0.007); no gender differences were seen for the left hand 2D : 4D digit ratio. Cohen's* d* indicating that effect size equals 0.01 for the left hand digit ratio and 0.03 for the right hand digit ratio in men versus women. In women, the left hand 2D : 4D digit ratio correlated significantly negative with scores on the subscale “communication” (*p* = 0.036; *r* = −0.142). In men, the left hand 2D : 4D digit ratio significantly positively correlated with the total AQ score (*p* = 0.042; *r* = 0.157) and with the subscale “attention switching” (*p* = 0.017; *r* = 0.182). No significant correlations were found between the right hand 2D : 4D digit ratio and any of the AQ subscales.

### 3.2. Dove-Type versus Hawk-Type Digit Ratios according to Gender

Regarding the* left* hand 2D : 4D digit ratio, in male hawk-type subjects a significant positive correlation of was found with the AQ subscale “attention to detail” (*p* = 0.014; *r* = 0.221), whereas in male dove-type subjects the left hand 2D : 4D digit ratio correlated significantly positive with both the total AQ score (*p* = 0.004; *r* = 0.408) and the subscales “attention switching” (*p* = 0.005; *r* = 0.389), “imagination” (*p* = 0.020; *r* = 0.328), and “attention switching” (*p* = 0.020; *r* = 0.324). In women, none of these correlations reached significance. Regarding the* right* hand 2D : 4D digit ratio, the only significant correlation for the right hand 2D : 4D digit ratio was found with the total AQ score in male dove-type subjects (*p* = 0.043; *r* = 0.274). All other correlations were not significant.

## 4. Discussion

Our findings confirm that for the population as a whole the 2D : 4D digit ratio does not significantly correlate with the total AQ score and its subscales. These correlations were also not significant when analyzing the data separately for dove-type and hawk-type subjects.

From these overall conclusions it may seem tempting to conclude that the 2D : 4D digit ratio is a poor biomarker of autistic traits in a healthy study population. However, the data do also show several interesting gender differences that should not be ignored. Whereas the left hand 2D : 4D digit ratio correlated significantly only with scores on the subscale “communication” in women, in men it significantly correlated with the total AQ score and the subscale “attention switching.” Moreover, in dove-type men both the left and right hand digit ratio significantly correlated with the total AQ score. Their left hand digit ratio further correlated significantly with the AQ subscales for “attention switching,” “imagination,” and “attention to detail.” In male hawk-type subjects, left hand digit ratios correlated significantly only with the subscale “detail oriented.” In women, these correlations were not significant.

The observed gender differences may be explained by the fact that ASD must be viewed as sexually dimorphic, just as the 2D : 4D digit ratio, with males on average having lower ratios than females [3, 21]. Low digit ratios correspond to high prenatal testosterone levels and relate to the expression of autistic traits and possible development of autism [10]. The latter also corresponds to the observation that ASD is strongly sex-dependent, that is, much more common in men than in women (with a male/female ratio of 4 to 1) [22]. Supportive evidence for the role of prenatal testosterone levels in the development of ADS also comes from a study that examined the expression of autistic traits in children with congenital adrenal hyperplasia (CAH). CAH is a genetic disorder causing excess adrenal androgen production, testosterone amongst others, already beginning in the fetal stage. Girls with CAH show masculinization of mood and behavior, relative to their unaffected siblings [23, 24]. Knickmeyer and Baron-Cohen identified girls with CAH to exhibit more autistic traits, compared to their unaffected siblings, and suggested it to be the result of higher prenatal androgen exposure [25].

Although several studies have suggested an association between prenatal levels of testosterone, the 2D : 4D digit ratio, and the risk of autism, other studies could not confirm these findings [10, 26–28]. For example, by comparing digit ratios of autistic children to those of their close relatives, Manning et al. predicted that high levels of prenatal testosterone do not invariably result in an autistic phenotype and that other factors may also precipitate an autistic phenotype [10]. In addition to the results of Manning and Taylor [8], Hönekopp [11] and Barona et al. [29] suggest high prenatal testosterone levels to predispose to autism only in individuals exposed to extremely high prenatal testosterone. The majority of our study population consisted of individuals with a 2D : 4D digit ratio that is as low as the ratio described by Hönekopp and Teatero and Netley to be a risk factor in developing ASD.

The absence of significant associations may further have been caused by differences in study design, that is, comparing 2D : 4D digit ratios of people with and without a formal ASD diagnosis versus associating the 2D : 4D digit ratio with severity of ADS trait measures in the general population [30].

A limitation of the current study is that data was collected from a relative healthy and young sample. University students are highly educated individuals who usually possess a more than average level of skills that are seen impaired in ADS, such as communication and attention switching. Recruitment of participants in a clinical setting would have provided qualitatively different data and stronger associations between the 2D : 4D digit ratio and specific aspects of autism spectrum disorder [31]. Therefore, future research concerning the association between ASD and the 2D : 4D digit ratio should include a broader age range, including the nonstudent population and patients.

## 5. Conclusion

Although the overall association between ADS and the 2D : 4D digit ratio was not significant, significant gender specific associations between the 2D : 4D digit ratio and specific autism traits were observed, which were stronger in men than in women. Future research should focus on these gender differences.

## Figures and Tables

**Table 1:** Description of participating subjects.

	Overall	Men	Women
Age	20.5 (2.3)	20.7 (2.4)	20.2 (2.1)
Total AQ score	102.2 (13.2)	105.1 (12.8)^*∗*^	99.9 (13.0)
Social insights and behavior	18.7 (4.4)	19.4 (4.7)^*∗*^	18.2 (4.2)
Attention switching	22.5 (3.9)	23.0 (4.0)^*∗*^	22.1 (3.9)
Communication	19.8 (3.8)	20.5 (3.9)^*∗*^	19.2 (3.6)
Imagination	20.2 (3.9)	21.1 (3.9)^*∗*^	19.5 (3.8)
Attention to detail	21.1 (4.5)	21.2 (4.1)	21.1 (4.8)
Left digit ratio	0.988 (0.036)	0.986 (0.034)	0.990 (0.036)
Right digit ratio	0.992 (0.034)	0.987 (0.034)	0.996 (0.034)

Mean (SD) values are presented for the total group and for men and women separately. Significant differences (*p* < 0.05) are indicated by *∗*.
